# The Genomic Characteristics of Potential Probiotics: Two *Streptococcus salivarius* Isolates from a Healthy Individual in China

**DOI:** 10.3390/microorganisms13030694

**Published:** 2025-03-20

**Authors:** Mingyue Sun, Qian Li, Feiran Zhang, Ding Yao, Wenhua Huang, Qingyu Lv, Hua Jiang, Decong Kong, Yuhao Ren, Shaolong Chen, Yongqiang Jiang, Peng Liu

**Affiliations:** 1 School of Basic Medical Sciences, Anhui Medical University, Hefei 230032, China; s1547162110@163.com (M.S.); yaof135@sina.com (D.Y.); 2 State Key Laboratory of Pathogen and Biosecurity, Academy of Military Medical Sciences, Beijing 100072, China; liqian_bime@163.com (Q.L.); huangwh1993@163.com (W.H.); lvqingyu2004@126.com (Q.L.); jhua76@126.com (H.J.); kongdecong-118@163.com (D.K.); www2354@126.com (Y.R.); kantchen@163.com (S.C.); 3 Division of Fifth, Peking University School and Hospital of Stomatology & National Center for Stomatology & National Clinical Research Center for Oral Diseases & National Engineering Research Center of Oral Biomaterials and Digital Medical Devices, Beijing 100081, China; zfr_jill@163.com

**Keywords:** *Streptococcus salivarius*, oral cavity, potential probiotic characteristics, genome sequencing

## Abstract

The isolation and characterization of novel probiotics from dairy products, fermented foods, and the gut have gained significant attention. In particular, *Streptococcus salivarius* shows promise for use in oral probiotic preparations. In this study, we isolated two strains of *S. salivarius*—S.82.15 and S.82.20—from the oral cavity of a healthy individual. These strains exhibited distinct antimicrobial profiles. We thoroughly assessed the morphology and growth patterns of both strains and confirmed auto-aggregation and hemolytic activity. Through comprehensive genomic analysis, we found notable strain differences within the same bacterial species isolated from the same individual. Notably, the presence or absence of plasmids varied between the two strains. The genome of S.82.15 spans 2,175,688 bps and contains 1994 coding DNA sequences (CDSs), while S.82.20 has a genome size of 2,414,610 bps, a GC content of 40.62%, and 2276 annotated CDSs. Both strains demonstrated antibacterial activity against Group A *Streptococcus* (GAS), *Micrococcus. luteus*, and *Porphyromonas gingivalis*. To investigate the antibacterial properties further, we identified a gene cluster of salivaricin 9 on the plasmid of S.82.20 and a *blp* gene family on the chromosomes of both S.82.15 and S.82.20. Moreover, the gene expression of the *blp* family was upregulated when the isolated strains were co-cultured with GAS.

## 1. Introduction

*Streptococcus salivarius* is one of the first bacterial species to colonize the oral cavity of newborns and persists as a predominant colonizer in the human oral cavity [1], which plays a crucial role in shaping the oral microbiome [2] and significantly impacts the ecological balance of the oral cavity [3]. It interacts with beneficial bacteria to stabilize microbial communities, stimulate the immune system, enhance local oral immunity, and help prevent periodontal diseases [4]. Additionally, *S. salivarius* produces antibacterial compounds, such as bacteriocins, which inhibit the growth of pathogenic bacteria and contribute to the stability of the oral microecological environment.

*S. salivarius* shows promising potential for oral probiotic applications. *S. salivarius* K12 and M18 represent the most commercially successful probiotics of this species to date, with global distribution. *S. salivarius* K12 produces the bacteriocins salivaricin A2 and salivaricin B [5], which inhibit the formation of cariogenic biofilms by interrupting the growth and glucosyltransferase production of *S. mutans* [6]. Furthermore, its bacteriocins exhibit inhibitory effects against Group A *Streptococcus* (GAS) [7]. M18 encodes salivaricin 9 (Sal9), and its cell-free supernatant demonstrates the growth suppression of *Pseudomonas aeruginosa* and *Klebsiella pneumoniae* [8]. These bacterium could provide new strategies for preventing and managing oral diseases such as halitosis [9], dental caries [10], and periodontitis [11] by modulating the oral microbiota.

The isolation and characterization of novel probiotics from dairy products [12], fermented foods [13], and the human gut [14] have garnered increasing attention [15]. Concurrently, probiotics are increasingly being utilized as novel therapeutic agents. For instance, *Lactobacillus reuteri* has been reported as an adjunctive treatment for periodontitis [16], while *L. casei* and *Bifidobacterium breve* have demonstrated efficacy in reducing *Candida* colonization in the oral cavity of elderly populations [17]. Clinically, randomized controlled trials have also elucidated the therapeutic significance of *S. salivarius* in oral health. For instance, *S. salivarius* has been shown to alleviate oral mucositis in patients undergoing radiotherapy for malignant head and neck tumors [18], prevent acute otitis media [19], and reduce the incidence of pharyngo-tonsillitis in pediatric populations [20].

*S. salivarius* exhibits robust antibacterial activity as a probiotic against various pathogenic bacteria. Safety assessments of *S. salivarius* K12 for oral disease management, including but not limited to toxicity, antibiotic resistance, and the production of deleterious metabolic by-products, alongside evaluations of its genetic stability [21], have been documented. Collectively, these evaluations demonstrate that *S. salivarius* is safe for human consumption.

Current research on *S. salivarius* has predominantly focused on *S. salivarius* K12 and M18 [4,5,6,8,22]. However, as a vast reservoir of bacteriocins, there is a critical need to isolate and sequence additional strains within this species. Comprehensive genomic analyses of diverse *S. salivarius* variants could reveal novel bacteriocin subtypes with distinct antimicrobial spectra. Such discoveries enable the targeted inhibition of emerging multidrug-resistant pathogens, thereby addressing the escalating global challenge of antibiotic resistance. This approach underscores the importance of expanding genomic exploration beyond characterized strains to unlock the full therapeutic potential of *S. salivarius*.

In this study, we isolated two distinct *S. salivarius* strains, S.82.15 and S.82.20, from the oral cavity of a healthy individual. These strains displayed genomic and antibacterial profile differences compared with well-documented *S. salivarius* K12. Both strains demonstrated antibacterial activity against *Streptococcus pyogenes* (Group A *Streptococcus*, GAS), *Micrococcus. luteus* (*M. luteus*), and *Porphyromonas gingivalis* (*P. gingivalis*). We predicted gene clusters responsible for producing salivaricins, specifically salivaricin 9 and the *blp* family of bacteriocins, which are encoded by the plasmid and chromosome, respectively. The null hypothesis (H_0_) states that there is no significant difference in gene expression between co-cultured group and control group. A transcriptome analysis of S.82.20 co-cultured with GAS further confirmed the upregulation of the genes encoding *blp* family bacteriocins. Our study expands the current diversity of *S. salivarius* strains. By integrating comprehensive genomic analyses with experimental validation, we identified key gene clusters potentially responsible for bacteriocin production. These findings enhance the therapeutic potential of *S. salivarius* as probiotics and propose a viable strategy to combat the global challenge of antibiotic resistance.

## 2. Materials and Methods

### 2.1. Subjects and Saliva Sampling

A healthy volunteer (aged 18–45 years; a non-smoker with no history of alcohol consumption) was recruited from the Fifth Dental Center of Peking University Hospital of Stomatology and identified as sample No. 82. This participant was screened to exclude dental caries, gingivitis, or antibiotic use within the preceding 3 months. Unstimulated whole saliva samples were collected using a sterile polypropylene tube via passive drooling or spitting. The samples were immediately placed on ice and processed within 2 h to preserve microbial integrity. Two distinct *S. salivarius* strains were isolated from sample No. 82. Isolation was performed using THB agar, followed by PCR identification.

### 2.2. Bacterial Strains and Culture

*S. salivarius* K12 was obtained from the commercially available oral probiotic product BLIS PROBIOTICS (Blis Technologies Limited, Dunedin, New Zealand). A single tablet was crushed and dissolved in 50 mL of phosphate-buffered saline (PBS). After complete dissolution, 50 μL of the suspension was inoculated into Todd Hewitt Broth (THB) medium and incubated aerobically at 37 °C for 24 h. Growth sedimentation patterns were quantitatively assessed through time-course OD_600_ measurements. Bacterial cultures incubated statically at 37 °C exhibited progressive supernatant clarification, with minimal OD_600_ variation in the upper liquid phase over 24 hr. In contrast, significant cellular sedimentation was observed at the culture base, forming a dense pellet. Vortex-induced resuspension of sedimented cells resulted in abrupt OD_600_ increases, confirming phase-dependent biomass redistribution. These metrics collectively demonstrate a sedimentation-dominant growth phenotype under static culture conditions. The indicator strains *M. luteus* ATCC 4698 and Group A *Streptococcus* (GAS) A909 were obtained from the American Type Culture Collection (ATCC). GAS was cultured in Luria–Bertani (LB) medium or THB medium, while *M. luteus* was cultured in TSB medium. Simultaneous antagonism tests were performed using a Columbia agar base supplemented with 5% defibrinated whole human blood. The agar plates were prepared fresh and used within 24 h to ensure optimal activity of the blood components. Simultaneous antagonism tests were conducted in triplicate to ensure statistical robustness and reproducibility.

### 2.3. Isolation and Identification of S. salivarius in Saliva Samples

#### 2.3.1. Isolation of *S. salivarius*

Saliva samples were serially diluted tenfold using PBS. Aliquots of the diluted samples were inoculated onto THB agar plates and incubated at 37 °C for 48 h under 5% CO_2_. Following incubation, at least 20 colonies were randomly selected for further isolation. Isolated bacterial colonies were inoculated into THB liquid medium and incubated at 37 °C for 24 h under 5% CO_2_. Based on colony morphology, medium-sized white colonies were selected for monoclonal purification. These colonies were inoculated into fresh THB liquid medium and incubated at 37 °C for 18–24 h to ensure pure cultures.

#### 2.3.2. Molecular Biology Identification of *S. salivarius*

Genomic DNA was extracted from overnight cultures of isolated strains by heating at 95 °C for 15 min in a water bath, followed by centrifugation to collect the DNA. The presence of *S. salivarius* was confirmed using colony PCR with specific primers: Sali-F (5′-GTGTTGCCACATCTTCACTCGCTTCGG-3′) and Sali-R (5′-CGTTGATGTGCTTGAAAGGGCACCATT-3′). PCR amplification was performed under the following conditions: initial denaturation at 94 °C for 10 min; 35 cycles of denaturation at 94 °C for 30 s, annealing at 55 °C for 30 s, and extension at 72 °C for 30 s; and a final extension at 72 °C for 7 min. The PCR products were separated by electrophoresis on a 1.5% agarose gel and visualized under UV light. The amplified fragments were subsequently verified by Sanger sequencing to confirm their identity.

### 2.4. Plasmid Extraction

Plasmid DNA was isolated from *S*. *salivarius* using the TIANprep Midi Plasmid Kit (TIANGEN, Beijing, China) according to the manufacturer’s instructions. Briefly, 10 mL of overnight bacterial culture was treated with lysozyme (20 mg/mL) and mutanolysin (5 U/mL) for enzymatic cell lysis. Alkaline lysis buffer and proteinase K were subsequently added to eliminate chromosomal DNA and proteins. Plasmid DNA was eluted in TE buffer and quantified using a NanoDrop spectrophotometer (Thermo Fisher Scientific, Waltham, MA, USA).

### 2.5. Detection of Salivaricin A2 and Salivaricin B

The *S. salivarius* strain was cultured in THB medium for 24 h at 37 °C, 5% CO_2_. Cells were harvested by centrifugation at 8000 rpm for 5 min and washed twice with 0.9% NaCl solution. The cell pellet was then suspended in a lysis buffer containing 20 mM Tris (pH 8.0), 2 mM Na_2_-EDTA, and 1.5% Triton X-100. The suspension was homogenized for 10 min to disrupt the cell walls. Subsequently, mutanolysin and lysozyme were added to the mixture and incubated at 37 °C for 10 min. Genomic DNA was isolated and purified using the TIANamp Bacteria DNA Kit (TIANGEN, Beijing, China) according to the manufacturer’s instructions. For the detection of salivaricin A2, the following primers were used: saliA-F (5′-TGATTGCCATGAAAAACTCAAAA-3′) and saliA-R (5′-CCACCAGCTACTTCCATAAGTTCTT-3′). Additionally, the primer pair saliB-F (5′-GTTGAGATTGAAGCAATGAATTCTCT-3′) and saliB-R (5′-ACCAGCACCAAGAACGTTATCA-3′) was employed to detect the *saliB* gene encoding salivaricin B production in *S. salivarius* strains.

### 2.6. Simultaneous Antagonism Assay

To assess the antimicrobial activity of *S. salivarius*, two strains were inoculated onto blood agar plates that had been previously seeded with indicator bacteria. The indicator bacteria utilized in this study to evaluate the antibacterial activity of *S. salivarius* included *M. luteus* ATCC 4698, *S. pyogenes* (GAS A909), and *P. gingivalis* BNCC 337441. *S. salivarius* K12, a known producer of salivaricin A2 and salivaricin B, was employed as a positive control, while THB medium was used as a negative control. Simultaneous antagonism tests were conducted in triplicate to ensure statistical robustness and reproducibility.

### 2.7. Scanning Electron Microscopy Images

*S. salivarius* was cultured for 18 h under the previously described conditions. Subsequently, 1 mL of the bacterial suspension was centrifuged at 8000 rpm and 4 °C for 5 min. The bacterial pellet was resuspended in 5% glutaraldehyde, which was used as the fixative, and incubated at 4 °C for at least 24 h. After fixation, the samples were removed from the glutaraldehyde solution and subjected to gradient dehydration using ethanol solutions of increasing concentrations (50%, 70%, and 100%), with each step lasting for 10 min. Following dehydration, the samples were transferred from the 100% ethanol solution to a supercritical dryer for critical point drying, which lasted approximately 1 h. To enhance electrical conductivity and reduce charge accumulation during scanning electron microscopy (SEM, Hitachi High-Tech, Tokyo, Japan) observation, the bacterial surfaces were coated with a thin layer of gold via sputtering. This process ensures clearer imaging by preventing the accumulation of static charges on the bacterial surfaces. After drying, the samples were removed from the critical point dryer, mounted onto SEM stubs using conductive tape, and coated with gold. The prepared samples were then subjected to SEM analysis.

### 2.8. The Next-Generation Sequencing and Third-Generation Sequencing

The next-generation sequencing was performed on the Illumina platform. The DNA samples qualified by electrophoresis were randomly sheared into fragments approximately 350 bps in length using a Covaris ultrasonicator (Covaris, Woburn, MA, USA). The processed DNA fragments were subsequently subjected to library preparation through sequential procedures including end repair, A-tailing, adapter ligation, purification, and PCR amplification using the NEBNext^®^ Ultra™ DNA Library Prep Kit for Illumina (NEB, Ipswich, MA, USA), following the manufacturer’s instruction.

Third-generation sequencing was performed on PacBio Sequel lle using the genomic DNA extracted as described above. The SMRTbell library was constructed using the SMRTbell™ Template Prep Kit 2.0 (Pacific Biosciences of California, Menlo Park, CA, USA). DNA samples passing electrophoretic quality control were fragmented into 6–20 kb segments using Covaris g-TUBE (Covaris, Woburn, MA, USA). Post-shearing purification was performed with AMPure PB Beads to concentrate the fragmented DNA for subsequent damage repair and end-repair processes. Adapters were ligated to the ends of each DNA fragment, followed by purification of SMRTbell templates using AMPure PB Beads (Pacific Biosciences of California, Menlo Park, CA, USA). Size selection was conducted using the Blue Pippin System to eliminate small insert SMRTbell templates, with subsequent library purification using AMPure PB Beads. The DNA damage repair process was then implemented, followed by an additional purification step employing AMPure PB Beads. The constructed library was quantified using Qubit fluorometric quantification, and insert fragment size distribution was verified using the Agilent 2100 Bioanalyzer system (Agilent Technologies, Santa Clara, CA, USA). Finally, sequencing was performed on the PacBio Sequel IIe platform (Pacific Biosciences of California, Menlo Park, CA, USA).

### 2.9. Data Processing and Genome Assembly

The low-quality reads of third-generation sequencing reads were filtered by the SMRT Link v8.0. The next-generation sequencing data were processed sequentially through FastQC (Version 0.11.9) for quality control assessment and Trimmomatic (version: 0.33) [23] for low-quality read trimming, employing default parameters for both tools.

The command line is as follows:

“fastqc -o ./ -- nogroup sample.1.fastq & sample.2.fastq”

“java -jar trimmomatic-0.33.jar PE \

Sample_R1.fq.gz Sample_R2.fq.gz \

Sample_Trim_paired_R1.fq.gz Sample_Trim_unpaired_R1.fq.gz \

Sample_Trim_paired_R2.fq.gz Sample_Trim_unpaired_R2.fq.gz \

ILLUMINACLIP:TruSeq3-PE.fa:2:30:10 \

LEADING:3 \

TRAILING:3 \

SLIDINGWINDOW:4:15 \

MINLEN:36”

The Canu assembler (version: 2.0) was employed to generate an initial genome assembly from the third-generation sequencing reads, providing a preliminary overview of the sample genome structure. Subsequently, Racon software (version 1.4.13) was utilized to conduct three rounds of error correction on the assembled genome, leveraging the third-generation sequencing data. This was followed by three additional rounds of error correction using Minimap2 (version 2.24) [24] alignment followed by Racon (version 1.22) with second-generation sequencing reads to refine the assembly and obtain the final, high-quality genome assembly.

The command line is as follows:

Canu Command:

canu -p sali -d sample genomeSize=2.4m -pacbio sample.fastq

Third-generation sequencing reads were aligned to the reference genome (GCA_000286295.1 SsalK12v1) using Minimap2 with the following command:

minimap2 -ax map-pb -t 8 Sali.ref.mmi sample.fasta.gz > map1.sam.

The initial assembly was polished with Racon using the following:

racon -t 8 sample.fasta.gz map1.sam sali.ctg.fa > Sali.sample.1.fa.

This polishing cycle (Minimap2 alignment followed by Racon correction) was iteratively repeated three times to refine assembly accuracy.

A final error correction was performed with Pilon by integrating short-read sequencing data using the following:

java -Xmx10G -jar pilon-1.22.jar --genome sali.fa --frags align_filter.bam --fix snp,indels --output pilon_polished --vcf & >pilon.log.

The resulting output represents the polished genome assembly.

### 2.10. Taxonomic Identification

We constructed a phylogenetic tree using the UBCG [25] (version 3.0) pipeline, which automates the extraction of core genes from genomic sequences and concatenates them for alignment. The workflow utilizes internally integrated MAFFT [26] (version 7.526) for multiple sequence alignment and FastTree [27] (version 2.1.11) for tree inference, ultimately generating a core gene-based phylogeny. Genomic FASTA files were first converted into BCG format files to store genome and strain metadata, followed by UBCG execution for tree construction. This study included 32 *S*. *salivarius* genomes retrieved from NCBI and one *Streptococcus pyogenes,* NCTC 12064, as the outgroup for rooting the tree. The relevant accession numbers and genome designations are provided in Table S1.

### 2.11. Genome Annotation and Functional Prediction

Coding genes in the newly sequenced genome were predicted using GeneMarkS (Version 4.28; https://genemark.bme.gatech.edu/GeneMark/genemarks.cgi, accessed on 11 December 2024). tRNA genes were identified using tRNAscan-SE (Version 1.3.1). Secondary metabolites, which are compounds synthesized by microorganisms during specific growth phases using primary metabolites as precursors. The antiSMASH program (Version 4.0.2) [28] was employed to predict secondary metabolite biosynthesis gene clusters within the genome. Predicted genes were annotated against the Non-Redundant Protein Database (NR) [29] and the Clusters of Orthologous Groups (COG) [30] database to identify gene functions and classify them into COG categories. Virulence factors were predicted using the Pathogen–Host Interactions (PHI) [31] database and the Virulence Factors Database (VFDB) [32]. Additionally, antibiotic resistance genes were identified using the Comprehensive Antibiotic Resistance Database (CARD) [33].

### 2.12. RNA Extraction

To compare the RNA expression levels between *S. salivarius* colonies growing under normal conditions and those in a competitive state, colonies from both growth conditions were collected. *S. salivarius* was initially cultured in liquid medium for 18 h. Subsequently, 5 µL of the bacterial suspension was spotted onto a blood agar plate and incubated overnight to form colonies, which were then harvested. For colonies in the competitive state, an indicator bacterium was first inoculated onto the surface of the blood agar plate before *S. salivarius* was introduced. Three biological replicates were included for each experimental condition to ensure statistical robustness and reproducibility. The collected colonies were washed three times with sterile PBS and centrifuged at 12,000 rpm for 2 min at 4 °C. The bacterial cells were then resuspended completely in 100 µL of TE buffer containing lysozyme. Total RNA was isolated and purified using the RNAprep Pure Cell/Bacteria Kit (TIANGEN, Beijing, China) according to the manufacturer’s instructions. The RNA was subjected to quality control using a Nanodrop spectrophotometer, ensuring purity criteria were met, with A260/A280 and A260/A230 ratios exceeding 2.0. RNA integrity was subsequently verified through fragment length analysis (2–5 kb) performed on an Agilent 2100 Bioanalyzer system.

### 2.13. RNA Sequencing and Transcriptome Analysis

The RNA sequencing library was constructed using the TIANSeq Stranded RNA-Seq Kit (TIANGEN, Beijing, China) according to the manufacturer’s instruction for the Illumina platform. The procedure encompassed the following steps: rRNA depletion, RNA fragmentation, first-strand cDNA synthesis, second-strand cDNA synthesis, double-stranded cDNA purification, end repair/dA-tailing, adapter ligation, library purification, size selection, and library amplification. RNA sequencing was performed using paired-end 150 bp reads. Raw sequencing data were subjected to quality control and filtering to remove low-quality reads. The assembled genome and corresponding annotation files were utilized for read alignment. Clean reads were mapped to the assembled genome mentioned earlier using Bowtie2 (version 2.4.4) [34] with a mismatch parameter of 2 and default settings for other arguments. The Rockhopper [35] graphical interface was employed to identify novel transcript regions. Required inputs included the genome annotation file in GenBank format (gbk), the assembled genome sequence (fasta), and aligned sequencing data. Differentially expressed genes (DEGs) between two experimental conditions were analyzed using the DESeq2 R package (v1.20.0) [36]. To control the false discovery rate (FDR), *p*-values were adjusted using the Benjamini–Hochberg method. A corrected *p*-value threshold of <0.05 was set to define statistically significant differential expression.

## 3. Results

### 3.1. Morphology of Isolated S. salivarius

**Figure 1:**
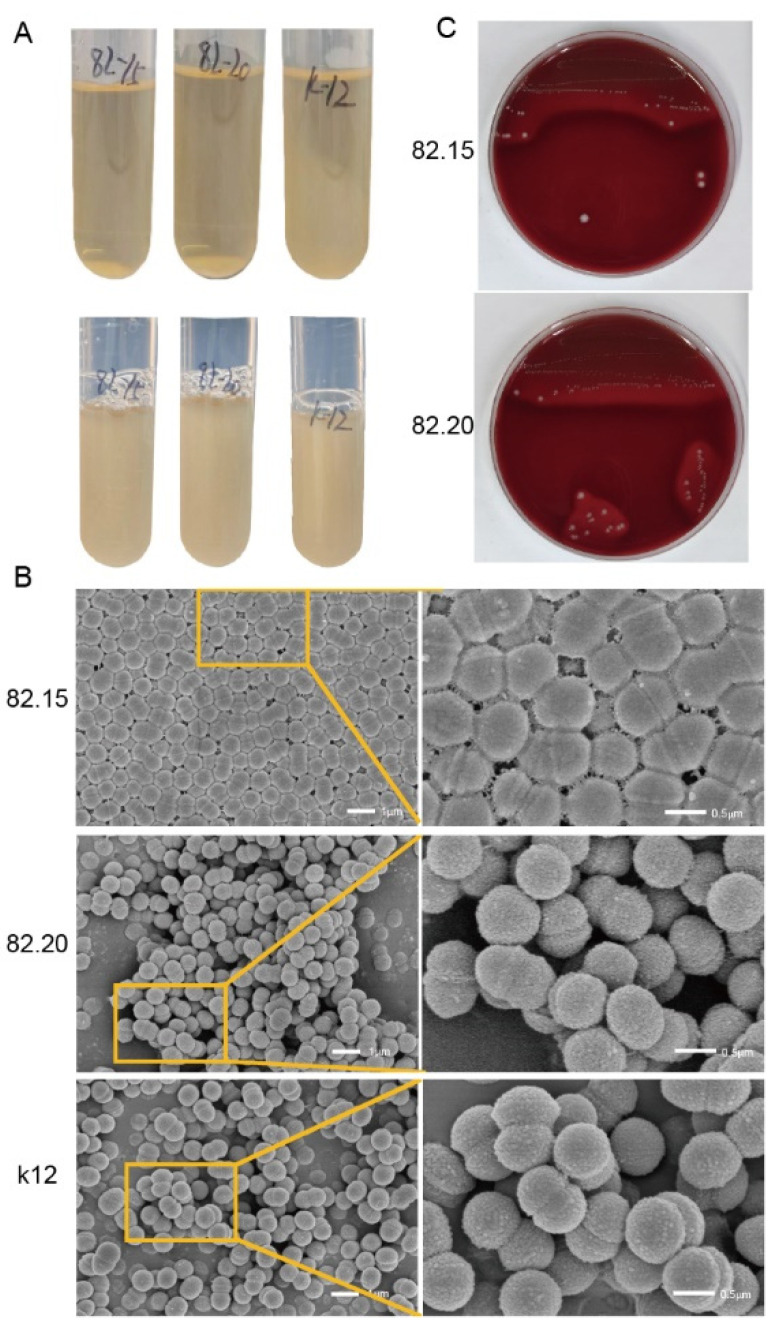
Characterization of two clinically isolated *S. salivarius* strains. (**A**) Morphology of *S. salivarius* grown in a liquid THB medium. (**B**) SEM images of S.82.15 (top), S.82.20 (middle), and *S. salivarius* K12. Scale bar, 1 μm (left) and 0.5 μm (right). (**C**) Alpha-hemolysis of the 2 isolated *S. salivarius* (top, S.82.15; bottom, S.82.20). All experiments were conducted in three biological replicates.

We isolated two *S. salivarius* strains from the oral cavity of a healthy individual, with initial identification confirmed by PCR. In liquid culture medium, the morphology of the isolated strains differed from that of the *S. salivarius* K12 strain. After 18 h of incubation at 37 °C with 5% CO_2_, the isolated strains exhibited sedimentary growth, which was proven by the OD_600_ measurements. The supernatant remained clear, while flocculent or granular precipitates settled at the bottom of the tube (Figure 1A, top). After shaking for 60 s, the suspension became homogeneous, but it rapidly precipitated and coagulated into clumps when left undisturbed (Figure 1A, bottom). In contrast, the *S. salivarius* K12 showed uniform turbidity in the liquid medium. The two *S. salivarius* strains isolated from the same individual exhibited consistent growth morphology in liquid culture. However, scanning electron microscopy (SEM) revealed morphological differences between the strains. As shown at the top of Figure 1B, S.82.15 differs from other *streptococci* by forming a tightly connected planar structure, with adjacent cells closely bound by substances secreted from the cell wall. These cells are not arranged in chains. In contrast, S.82.20 (Figure 1B, middle) exhibits less cohesive cell connections, and no distinct chains are formed between cells, except during the division phase. This morphology resembles that of the K12 strain (Figure 1B, bottom). We also examined the hemolytic activity of the isolated strains. As shown in Figure 1C, both S.82.15 and S.82.20 and the K12 strain exhibited hemolysis activity on blood agar. Overall, the two *S. salivarius* strains isolated from the same individual displayed similar growth patterns and hemolytic activity in both liquid and solid culture media. However, their SEM morphologies differed, suggesting genomic variations among these strains.

### 3.2. Antibacterial Activity of the Isolated Strains

To test the antibacterial activity of the two isolated strains, we conducted simultaneous antagonistic experiments using the indicator bacteria GAS and *M. luteus* under both aerobic and anaerobic conditions. The inhibitory effects of the isolated strains varied depending on the culture conditions. Under aerobic conditions, S.82.20 exhibited stronger inhibition of *M. luteus* compared to the K12 strain. S.82.15 produced the largest inhibition zones against GAS, followed by strain S.82.20, while the K12 strain showed the smallest inhibition zones. Under anaerobic conditions, the inhibitory effects of S.82.15 and S.82.20 on *M. luteus* were comparable to the K12 strain. The inhibitory effects of S.82.15, S.82.20, and the well-characterized K12 strain against indicator bacteria were systematically evaluated under distinct culture conditions (Table 1). Notably, the isolates exhibited superior antimicrobial efficacy compared to K12 under aerobic conditions, as quantified by zones of inhibition. 

**Figure 2:**
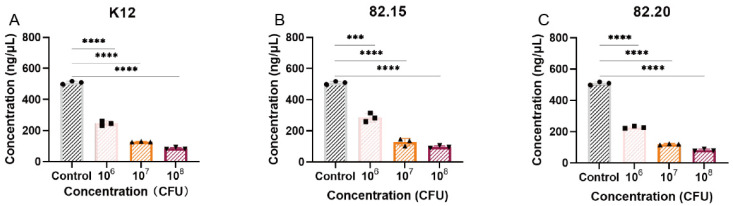
Co-culture of isolated *S. salivarius* and *P. gingivalis*. From left to right: *S. salivarius* K12 (**A**), S.82.15 (**B**), and S.82.20 (**C**). All samples are co-cultured with *P. gingivalis* (*n* = 3 biologically independent replicates, mean ± S.D.). *** *p* < 0.001, **** *p* < 0.0001 in two-tailed *t*-tests.

As *S. salivarius* plays a crucial role as a probiotic in the oral cavity, we also examined its antibacterial activity against the oral pathogen *P. gingivalis*. Due to the difficulty of observing *P. gingivalis* in simultaneous antagonistic experiments, we quantified bacterial counts under co-culture conditions to compare inhibitory effects. As shown in Figure 2, after 3 days of co-culturing *P. gingivalis* with *S. salivarius*, there was a significant reduction in *P. gingivalis* viability compared to single-culture growth. When co-cultured at a concentration of 10^6^ CFUs, strains K12, 82.15, and 82.20 resulted in average reductions of 51.63%, 43.94%, and 55.25% in *P. gingivalis* nucleic acid concentration, respectively. As the concentration of *S. salivarius* increased in the co-culture, the reduction in *P. gingivalis* nucleic acid concentration became more pronounced. The simultaneous antagonistic and co-culture experiments demonstrated that the *S. salivarius* strains isolated from the same individual exhibited differential inhibitory effects against various indicator bacteria, depending on the culturing conditions.

### 3.3. Genomic Analysis of S.82.15 and S.82.20

The results from the simultaneous antagonistic assays, conducted under varying conditions with different indicator bacteria and culture environments, revealed distinct antibacterial profiles for strains 82.15 and 82.20. To further investigate their antibacterial components, we performed third-generation sequencing on both strains and constructed their complete genome maps. The relevant genomic data are summarized in Table 2. The genome of strain 82.15 spans 2,175,688 base pairs (bps) and contains 1994 CDSs, of which 1960 genes were matched to the nr database, including 220 hypothetical proteins. In contrast, the genome of strain 82.20 is 2,414,610 bps in length, with a GC content of 40.62%. It encodes 2276 protein-coding sequences, 345 of which are hypothetical proteins. The Cluster of Orthologous Groups (COG) annotations for strains 82.15 and 82.20 are provided in Table 3. A higher proportion of genes in strain 82.20 were classified under categories such as replication, recombination and repair, defense mechanisms, and mobilome: prophages and transposons. In comparison, strain 82.15 contained a significantly higher number of non-coding RNAs (ncRNAs). Specifically, tRNAscan-SE software predicted 78 tRNA regions in strain 82.15, while only 58 were identified in strain 82.20 (Table 4).

**Figure 3:**
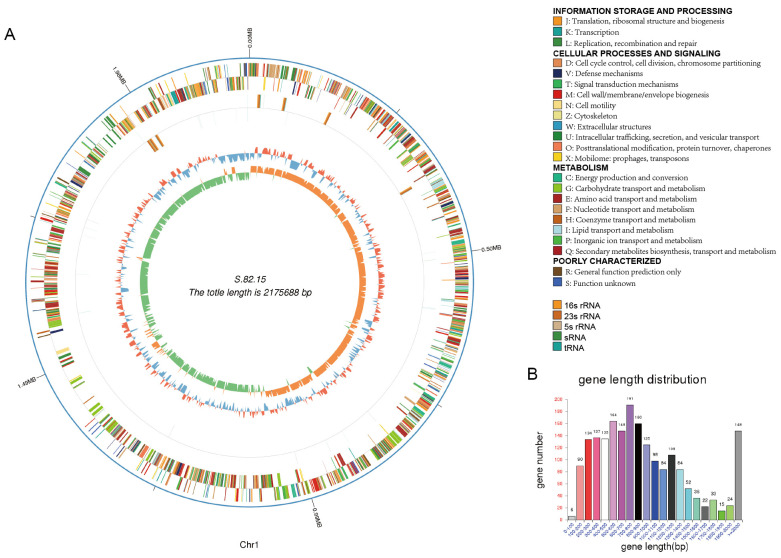
Circular view and gene length distribution of the *S. salivarius* S.82.15 genome. (**A**) From the outermost to the innermost ring: genomic sequence position coordinates, gene functional annotation results, non-coding RNAs (ncRNAs), genomic GC contents (the inward blue sections indicate lower-than-average GC content regions, while the outward red sections indicate higher-than-average GC content regions), genomic GC skew values (the inward green sections denote regions where the G content is lower than the C content and vice versa for the outward orange sections). Circus plots are generated by Proksee [37]. (**B**) Gene length distribution of *S. salivarius* S.82.15.

**Figure 4:**
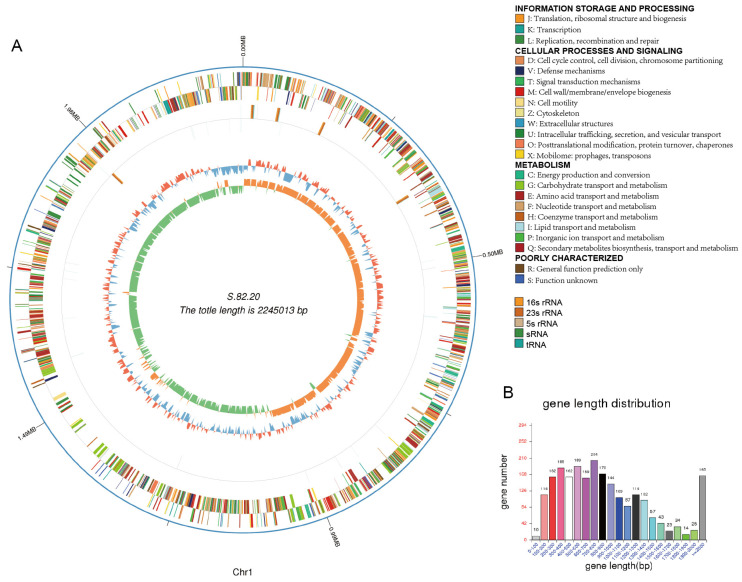
Circular view and gene length distribution of the *S. salivarius* S.82.20 genome. (**A**) From the outermost to the innermost ring: genomic sequence position coordinates, gene functional annotation results, non-coding RNAs (ncRNAs), genomic GC contents (the inward blue sections indicate lower-than-average GC content regions, while the outward red sections indicate higher-than-average GC content regions), genomic GC skew values (the inward green sections denote regions where the G content is lower than the C content and vice versa for the outward orange sections). Circus plots are generated by Proksee [37]. (**B**) Gene length distribution of *S. salivarius* S.82.15.

**Figure 5:**
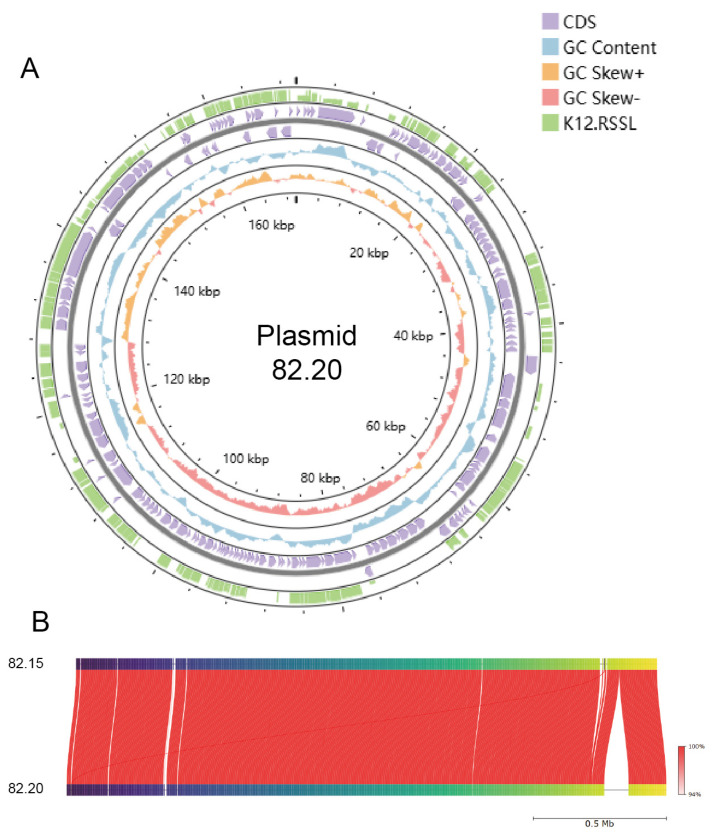
Genomic analysis of the strain 82.20 plasmid. (**A**) Circular view of the *S. salivarius* S.82.20 plasmid genome. From the outermost to the innermost ring: identity between the K12 RSSL plasmid and the S.82.20 plasmid CDSs (column heights represent the identities), gene functional annotation results encoding on the (+) strand and the (−) strand, genomic GC content (the inward sections indicate lower-than-average GC content regions, while the outward sections indicate higher-than-average GC content regions), genomic GC skew values (the inward sections denote regions where the G content is lower than the C content and vice versa for the outward sections). Circus plots are generated by Proksee [37]. (**B**) Collinearity analysis of chromosome genome between S.82.15 and S.82.20.

We constructed genomic maps for strains 82.15 and 82.20, which are shown in Figure 3 and Figure 4, along with the gene length distribution. Additionally, we completed the plasmid assembly of *S. salivarius* 82.20, as depicted in Figure 5A. The assembled plasmid is 169,597 bps in length with a GC content of 34.66%. This plasmid encodes 173 genes, 162 of which match homologous sequences in the NCBI nr database. The third-generation sequencing results indicate the absence of plasmids in strain 82.15. Furthermore, within the genomic region from positions 2,013,229 to 2,103,636 of strain 82.20, we did not identify any sequences encoding proteins with high similarity to those in strain 82.15. The collinearity analysis of chromosome genomes between strains 82.15 and 82.20 shows genome structural variation, which is illustrated in Figure 5B.

Additionally, we conducted a comparative analysis of chromosomal and plasmid average nucleotide identity (ANI) among S.82.15, S.82.20, and other plasmid-bearing *S. salivarius* strains, as summarized in Table 5. The chromosomal ANI values ranged from 94.99% to 96.57%, while plasmid ANI exhibited marked variation between 92.47% and 95.58%. The observed differences in genome size coupled with ANI variations suggest significant functional divergence between these isolates and established reference strains.

**Figure 6:**
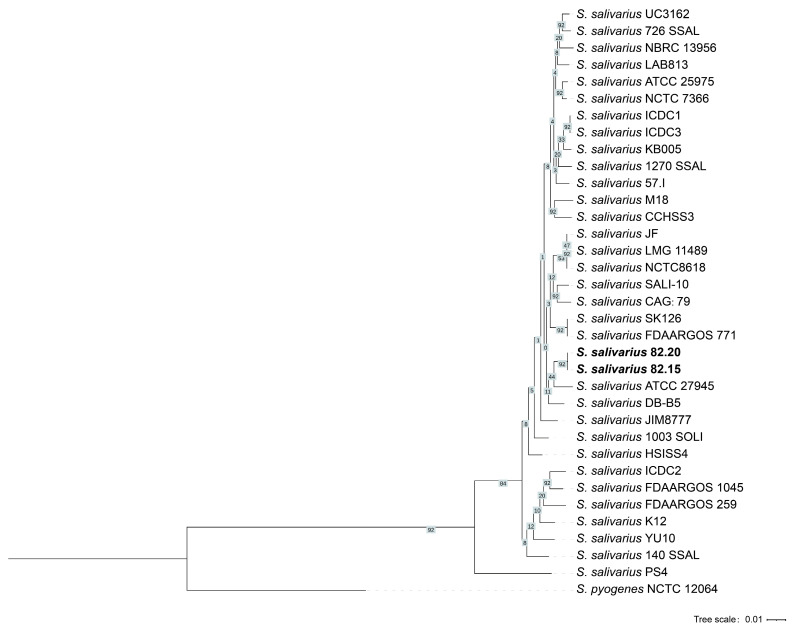
Phylogenetic trees generated by UBCG. The tree is rooted using *Streptococcus pyogenes* NCTC 12064. Bootstrap values are shown around each internode. *S. salivarius* S.82.15 and S. 82.20 are denoted in bold type.

To elucidate the phylogenetic relationship between S.82.15 and S.82.20 within *S. salivarius*, we performed a core genome-based phylogenetic analysis. The UBCG (Up-to-date Bacterial Core Gene) pipeline was employed to automatically extract and concatenate core genes, followed by multiple sequence alignment using MAFFT and maximum likelihood tree reconstruction with FastTree. The resulting phylogeny, rooted with *S. pyogenes* NCTC 12064 (Figure 6), robustly placed both S.82.15 and S.82.20 within the *S. salivarius* clade, supported by strong bootstrap values. Furthermore, these isolates exhibited substantial phylogenetic divergence from the well-characterized K12 and M18 clades, consistent with their distinct genomic profiles.

### 3.4. Detection of Potential Antibacterial Proteins

Salivaricins, which are encoded by *S. salivarius*, are primarily located on plasmids. These include salivaricin A2, salivaricin B, salivaricin G32, and salivaricin 9. To identify the inhibitory molecules in the *S. salivarius* strains isolated from healthy individuals in this study, we conducted a detailed comparative analysis of the annotated plasmids. We performed a whole-plasmid sequence alignment, with the top five ranked plasmids presented in Table 6. The plasmid from *S. salivarius* strain 82.20 showed high identity values (above 90%) when compared with known plasmids; however, the query coverage was low, indicating that the plasmid differs from previously reported ones. We analyzed the plasmid for secondary metabolite production using antiSMASH, which identified one secondary metabolite: a class II lanthipeptide. This metabolite exhibited 72% similarity to the known salivaricin 9 gene cluster.

**Figure 7:**
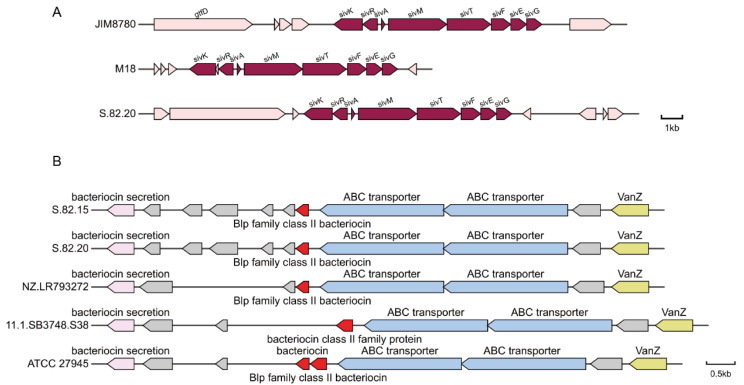
Gene clusters of salivaricin 9 (**A**) and *blp* family bacteriocin (**B**).

To further explore the plasmid’s potential, we compiled a local database of 4924 sequences, including 226 probiotic-related feature sequences from previous studies and all protein sequences encoded as “salivaricins” from NCBI. By aligning the proteins encoded by the *S. salivarius* S.82.20 plasmid with this database, we identified a 100% match between the amino acid sequence of *S. salivarius* S.82.20.GM000146 and the *sivA* (structural gene for salivaricin 9 production) sequence. Table 7 lists the identity values of the *sivK*, *sivR*, *sivA*, *sivM*, *sivT*, *sivF*, *sivE*, and *sivG* gene sequences between the *S. salivarius* S.82.20 plasmid and the reference plasmid. We compared the salivaricin 9 gene cluster of S.82.20 with the previously reported salivaricin 9 clusters encoded by the chromosome of *S. salivarius* JIM8780 and the plasmid of *S. salivarius* M18 as illustrated in Figure 7A. The results demonstrated that the gene cluster of S.82.20 exhibited identical sequence homology and genetic organization to both salivaricin 9 variants. Both the biosynthetic gene cluster (BGC) analysis and peptide BLAST (version 2.16.0) results confirm that the presence of salivaricin 9 is responsible for the antibacterial effect of *S. salivarius* S.82.20 against various indicator bacteria.

Strain 82.15 does not contain plasmids but still exhibits antibacterial activity. This suggests that antibacterial components may be encoded on its chromosome. Through the comparison and analysis of annotated CDSs, we identified three bacteriocin-related genes in *S. salivarius* S.82.15. These include a *blp* family class II bacteriocin (encoded by GM001626) and a class IIb bacteriocin (encoded by GM001828 and GM001829). These bacteriocin genes were also found in the chromosomal genome of *S. salivarius* S.82.20. The gene clusters for the *blp* family class II bacteriocin in S.82.15 and S.82.20, two closely related strains, are illustrated in Figure 7B. We further analyzed the *blp* family class II bacteriocin gene clusters encoded by another three *S. salivarius* isolates—NZ.LR793272, 11.1.SB3748.S38, and ATCC 27945—and compared them with the S.82 strain. The results revealed conserved gene arrangements across all isolates; however, divergent insertion fragments were identified between homologous genes.

In comparison with local databases and secondary metabolite gene cluster analyses, we confirmed that *S. salivarius* S.82.20 contains plasmid-encoded salivaricin 9-related genes, along with chromosomally encoded class IIb bacteriocins and *blp* family class II bacteriocins, which likely contribute to its antibacterial effects. In contrast, *S. salivarius* S.82.15 harbors only the chromosomally encoded class IIb bacteriocins and *blp* family class II bacteriocins, which may be responsible for its antibacterial activity.

### 3.5. Transcriptomic Screen of S.82.20 Against GAS

**Figure 8:**
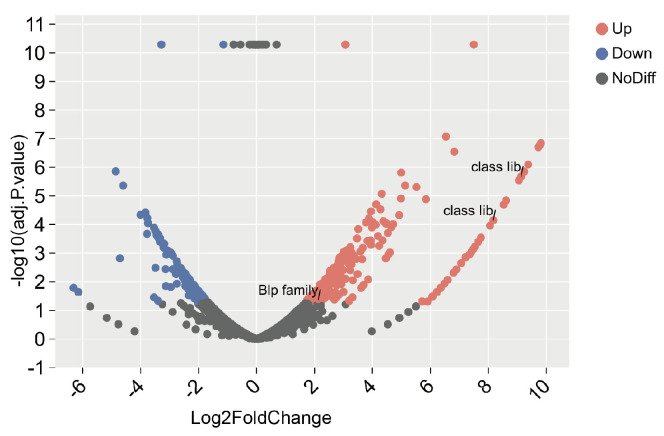
Transcriptomic analysis of S.82.20 against Group A *streptococcus*. Volcano plot displaying differentially expressed genes between co-cultured GAS and S.82.20 vs. normal cells in the chromosome. Blue dots represent downregulated genes, and red dots represent upregulated genes.

To determine whether the observed antibacterial effects are mediated by specific antibacterial factors, we co-cultured S.82.20 with GAS and performed transcriptomic analysis on *S. salivarius* under co-culture conditions. The results revealed that the expression levels of *blp* family and class IIb bacteriocins were significantly upregulated in *S. salivarius* S.82.20 during co-culture. As shown in Figure 8, the Log2FoldChange values for GM001981 and GM001982 (encoding class IIb bacteriocin) were 9.13 and 8.17, respectively. The Log2FoldChange value for GM001793 (encoding *blp* family bacteriocin) was 2.13. These findings suggest that the upregulation of *blp* family and class IIb bacteriocins contributes to the inhibition of GAS growth during co-culture with *S. salivarius* S.82.20.

We further analyzed plasmid expression dynamics under co-culture conditions. The most significant expression change was observed in S.82.20_GM000024 (log2FoldChange = 7.53, *p* = 7.45 × 10^−7^), annotated as a DUF2570 family protein. This sequence exhibits 97.19% identity to a homolog in Streptococcus salivarius and remains poorly characterized in this species. The NCBI database describes this protein as a “DUF2570 domain-containing protein with similarity to Haemophilus phage lytic protein Rz”, implying potential lytic functionality. Further investigation, including in vitro functional assays, is required to validate its role and mechanisms.

### 3.6. Toxicity and Biosafety Assessment

To assess the in vivo safety of *S. salivarius*, we evaluated its virulence and antibiotic resistance profiles. We compared the genomes of *S. salivarius* S.82.15 and S.82.20 to the PHI (Pathogen–Host Interaction) database and the VFDB (Virulence Factors Database) to identify potential pathogenic factors. We also compared the genomes to the CARD (Comprehensive Antibiotic Resistance Database) to screen for antibiotic resistance genes. In accordance with previously published criteria for resistance gene identification [21], open reading frames (ORFs) were required to exhibit “perfect” sequence matches (100% identity to CARD reference sequences) for classification as resistance genes. Notably, none of the ORFs from strains S.82.15 or S.82.20 met this stringent threshold. For virulence factor assessment, we applied the same study’s standards, employing a 90% identity threshold and a statistical cutoff of E-values 10^−5^. Comparative analysis identified 23 coding sequences (CDSs) across both databases that satisfied these criteria. To further contextualize these findings, we cross-referenced these sequences with other *S. salivarius* genomes. All 23 CDSs were identical to *S. salivarius* DB-B5 [21] or K12 [38], which already established safety credentials, suggesting that sequences do not confer virulence. Collectively, these results demonstrate that both isolated strains lack detectable virulence-associated genes and clinically relevant antibiotic resistance determinants, supporting their safety profile for potential applications.

## 4. Discussion

*S. salivarius*, a probiotic strain capable of producing bacteriocins, offers a promising strategy to address the escalating challenge of bacterial antibiotic resistance—a critical threat to global public health. Furthermore, it presents an alternative therapeutic approach for oral diseases that avoids disruption of the oral microenvironment, distinguishing it from conventional natural product-derived treatments [39]. Probiotics have been isolated from various environments, such as the gut [40] and neonatal feces [41]. These probiotics can produce bacteriocins that inhibit the growth of pathogenic bacteria, including *Escherichia coli*, *Staphylococcus aureus*, and others. In the oral environment, *S. salivarius* is one of the most common oral probiotics. The salivaricin it produces can inhibit the growth of GAS and *S. pneumoniae* [40].

In this study, we isolated *S. salivarius* from the oral cavity of a healthy individual to identify strains with distinct antibacterial properties. From sample No. 82, we successfully isolated five *S. salivarius* strains and conducted phenotypic and antibacterial assays. By comparing the inhibitory effects of these isolates with the K12 strain on GAS, *M. luteus*, and *P. gingivalis*, we identified two distinct *S. salivarius* strains from this healthy individual (sample No. 82), specifically strains 82.15 and 82.20. These strains exhibited superior antibacterial activity against the indicator pathogens compared to *S. salivarius* K12. Previous research has shown that *S. salivarius* K12, a commercially available probiotic, produces plasmid-encoded salivaricin A2 and salivaricin B, which inhibit pathogen growth in dental biofilms [5]. These bacteriocins also help maintain the physiological balance of the oral microbiome, playing a crucial role in dental biofilm homeostasis [42]. At the outset of our study, we hypothesized that the two *S. salivarius* strains from sample No. 82 might harbor salivaricin A2 and salivaricin B. However, using specific primers, we found that these two strains did not possess these salivaricins. We then examined the phenotypes of these strains in both liquid and solid media, noting significant differences in their growth patterns compared to the representative K12 strain. In liquid culture, *S. salivarius* strains 82.15 and 82.20 exhibited a sedimented growth pattern, with visible granules and flocculent material in the medium. SEM images revealed that *S. salivarius* S.82.15 cells were tightly interconnected by substances secreted from the cell wall, including cell wall-associated fimbriae and fibrils [43].

Benoit Couvigny and colleagues have previously reported that *S. salivarius* often undergoes auto-aggregation, a process facilitated by cell wall-secreted proteins, which promotes adhesion to other bacteria of the same species [44]. Probiotic organisms typically protect against infections through auto-aggregation or co-aggregation with pathogens. Auto-aggregation enables competitive exclusion and displacement of pathogens, while co-aggregation brings the probiotic bacteria’s Type VI secretion systems into closer proximity to pathogens, enhancing the release of antimicrobial substances [45]. Thus, the auto-aggregation observed in *S. salivarius* S.82.15 likely contributes to its antibacterial properties.

To identify the antibacterial factors in these two strains, we performed third-generation whole-genome sequencing and constructed complete genome maps for both. In addition to its chromosomal genome, S.82.20 harbors a 169-kilobase mega-plasmid. Previous studies indicate that salivaricin in *S. salivarius* is primarily plasmid-encoded [46]. Therefore, we first examined sequence similarities between the plasmids of S.82.20 and K12. The average nucleotide identity (ANI) between the two plasmids was only 92.47%, revealing substantial sequence divergence. Next, we annotated the plasmid-encoded proteins and used antiSMASH [28] to predict secondary metabolite biosynthetic gene clusters. Comparative analysis confirmed that the S.82.20 plasmid harbors a salivaricin 9 gene cluster with a structure and sequence identical to previously characterized clusters [47,48]. Salivaricin 9 belongs to the Type AII lantibiotic family and is encoded by genes responsible for its production, modification, transport, and regulation. The regulatory protein SivR detects variations in salivaricin levels inside and outside the cell, while SivK modifies the inactive precursor peptide post-translation. Additionally, the *sivEFG* gene encodes proteins that provide self-immunity by expelling the active lantibiotic, thereby preventing pore formation and disruption of cell wall synthesis [49]. Previous research by Abdelahhad Barbour demonstrated that salivaricin 9 penetrates the cytoplasmic membrane, induces pore formation, and ultimately leads to cell death [48].

Unlike S.82.20, strain 82.15 does not carry a plasmid but still exhibits antibacterial activity, suggesting that its chromosome encodes salivaricin or other bacteriocins. By comparing annotated proteins with known databases, we identified chromosomal gene clusters in both S.82.15 and S.82.20 encoding *blp* family proteins and class IIb bacteriocins. Previous studies have reported *blp* family gene clusters in *S. pneumoniae* [50,51] and *S. thermophilus* [48]. Additionally, Gaia Vertillo Aluisio identified a *blp* bacteriocin-encoding BGC and two class IIb bacteriocins in *S. salivarius* 24SMBc, which exhibited antibacterial activity against *S. pneumoniae* and *S. pyogenes*. In vitro assays demonstrated that 24SMBc significantly inhibited adhesion and promoted co-aggregation against *S. pneumoniae*. Furthermore, co-culture experiments with GAS revealed that the expression of these genes increased, confirming the functional role of class IIb bacteriocins and *blp* family gene clusters in GAS inhibition.

To date, the salivaricins primarily reported in the literature include salivaricin A (A1, A2, A3, A4, and A5), salivaricin B [5], salivaricin 9 [47,48], salivaricin G32 [52], salivaricin E [53], and salivaricin D [54]. Different salivaricins exhibit distinct antibacterial mechanisms and antimicrobial spectra. SalA and SalB inhibit bacterial growth by suppressing cell wall synthesis, demonstrating potent activity against GAS, *M. luteus*, *S. pneumoniae*, and *Streptococcus sanguinis*. In contrast, Sal9 and SalG32 exert their antibacterial effects through pore formation, effectively inhibiting the growth of GAS, *Enterococcus* spp., *Listeria monocytogenes*, and *Bacillus* spp. The antibacterial mechanisms of SalE and SalD remain uncharacterized; however, their antimicrobial spectra primarily target *S. mutans*, GAS, and *S. pneumoniae*. In this study, the isolated strains S.82.15 and S.82.20 encoding Sali9, class IIb bacteriocins, and blp family gene clusters not only exhibited inhibitory activity against GAS and *M. luteus* but also demonstrated novel efficacy in suppressing the growth of the oral pathogenic bacterium *P. gingivalis*.

In this study, we systematically examined the morphology and growth characteristics of both strains and confirmed their auto-aggregation and hemolytic activity. A thorough genomic analysis revealed strain-specific differences even among bacterial isolates from the same individual, including variations in plasmid carriage. To characterize their antibacterial properties, we predicted potential salivaricin-encoding gene clusters and confirmed the presence of salivaricin 9 and the *blp* family. Compared to the commercially available *S. salivarius* K12 strain, the two isolated strains (S.82.15 and S.82.20) exhibited distinct salivaricin-encoding and antimicrobial profiles. The K12 strain harbors plasmid-encoded *salA2* and *salB*, whereas S.82.20 carries a chromosomally located *blp* family cluster and a putative class IIb bacteriocin, along with a plasmid-encoded salivaricin 9. Furthermore, comparative genomic analysis of S.82.15 and S.82.20 with other plasmid-bearing *S. salivarius* strains revealed substantial divergence in both chromosomal and plasmid sequences. The chromosomal average nucleotide identity (ANI) ranged between 94.99% and 96.57%, while plasmid ANI varied markedly from 92.47% to 95.58%. However, this study has several limitations. Currently, the potential antibacterial protein was predicted solely through bioinformatic approaches, and its activity has not been validated by protein purification or in vitro assays. To address these limitations, future studies will focus on the following aspects to further investigate these two strains. Firstly, the *blp* family gene cluster identified in the chromosomal region exhibits structural divergence from known homologs, and whether this variation influences its antibacterial efficacy remains uncharacterized. Secondly, strain 82.15 demonstrates a markedly distinct inhibitory effect against *M. luteus* under anaerobic versus aerobic conditions. Intriguingly, we also observed that the hemolytic activity of the isolated strains is significantly enhanced under anaerobic conditions compared to aerobic environments. Previous reports suggest that the hemolytic activity of *S. salivarius* is linked to bacteriocin production [55]. We hypothesize that strain S.82.15’s ability to inhibit *M. luteus* is related to bacteriocin production associated with this hemolytic activity. Further experiments are required to test these hypotheses.

## 5. Conclusions

In this study, we isolated two genetically distinct *S. salivarius* strains from the oral cavity of a single healthy individual. The two strains exhibited differences in genome size and plasmid carriage. Both strains demonstrated antibacterial activity against GAS, *M. luteus*, and *P. gingivalis*. Collectively, analyses of secondary metabolites and transcriptomic data revealed that chromosome-encoded class IIb bacteriocins and *blp* family gene clusters play a pivotal role in mediating the observed antibacterial effects.

## Figures and Tables

**Table 1:** Simultaneous antagonistic activity against different indicator strains.

	GAS ^a^		*M. luteus* ^a^	
	Aerobic	Anaerobic	Aerobic	Anaerobic
82.15	+++	++	−	++
82.20	++	++	++	++
K12	+	++	+	++

^a^, levels of inhibition. “−” for no inhibition, “+” to “+++” for incremental inhibitory effects.

**Table 2:** Genomic information of S.82.15 and S.82.20.

	S.82.15	S.82.20
Genome Size	2,175,688	2,414,610
Gene Number	1994	2276
Gene Length	1,900,044	2,119,581
GC Content	40.93	40.62
% of Genome (Genes)	87.33	87.78
Gene Average Length	953	931
Gene Internal Length	275,644	295,029
Gene Internal GC Content	34.93	33.99
% of Genome (Internal)	12.67	12.22

**Table 3:** COG of S.82.15 and S.82.20.

Functional Class	Class Description	S.82.15	S.82.20
C	Energy production and conversion	46	47
D	Cell cycle control, cell division, and chromosome partitioning	30	32
E	Amino acid transport and metabolism	175	177
F	Nucleotide transport and metabolism	87	87
G	Carbohydrate transport and metabolism	91	93
H	Coenzyme transport and metabolism	92	92
I	Lipid transport and metabolism	54	54
J	Translation, ribosomal structure, and biogenesis	201	203
K	Transcription	109	115
L	Replication, recombination, and repair	90	105
M	Cell wall/membrane/envelope biogenesis	113	117
N	Cell motility	14	16
O	Post-translational modification, protein turnover, and chaperones	58	59
P	Inorganic ion transport and metabolism	72	73
Q	Secondary metabolites biosynthesis, transport, and catabolism	12	12
R	General function prediction only	110	111
S	Function unknown	76	78
T	Signal transduction mechanisms	82	86
U	Intracellular trafficking, secretion, and vesicular transport	25	25
V	Defense mechanisms	56	65
W	Extracellular structures	6	6
X	Mobilome: prophages and transposons	29	42
Z	Cytoskeleton	1	1

**Table 4:** Predicted ncRNA via tRNAscan of S.82.15 and S.82.20.

Sample ID	Type	Number	Average Length (bps)	Total Length (bps)
S.82.15	tRNA	78	75	5891
S.82.15	5s	7	136	949
S.82.15	16s	7	1536	10,753
S.82.15	23s	7	2899	20,293
S.82.15	sRNA	10	108	1087
S.82.20	tRNA	58	75	4379
S.82.20	5s	5	132	660
S.82.20	16s	5	1537	7684
S.82.20	23s	5	2901	14,504
S.82.20	sRNA	12	121	1457

**Table 5:** Average nucleotide identity of S.82. and other plasmid-bearing *S. salivarius*.

Strain	Assembly Accession	Genome Size (bps)	ANI of S.82.15 Chromosome (%)	ANI of S.82.20 Chromosome (%)	Plasmid Size (bps)	ANI of S.82.20 Plasmid (%)
M18	GCA_000225385.2	2,142,944	95.64	95.45	183,037	95.98
K12	GCA_000286295.1	2,241,314	95.02	94.99	185,045	92.47
SALI-10	GCA_022936265.1	2,096,969	96.55	96.57	164,439	93.2
ATCC 25975	GCA_002094975.1	2,199,793	95.90	95.79	126,555	93.24
LAB813	GCA_008305695.1	2,242,557	95.87	95.77	183,700	92.86

**Table 6:** Blast results of the S.82.20 plasmid.

Query ID	Subject ID	Query Cover (%)	Identity (%)	Description
S.82.20.Plas	NZ_MN480762.1	62.23	93.06	NU10 plasmid pSsal-NU10
S.82.20.Plas	NZ_CP018188.1	63.08	95.33	ICDC2 plasmid
S.82.20.Plas	NZ_CP040803.1	50.4	94.93	LAB813 plasmid pSAL813
S.82.20.Plas	NZ_CP090008.1	50.4	94.92	SALI-10 plasmid pSaLI10
S.82.20.Plas	NZ_CP015284.1	46.84	94.29	ATCC 25975 plasmid unnamed

**Table 7:** Blast results of the S.82.20 salivaricin 9 gene cluster.

Gene ID	Gene Name	Accession Number	Identity	E Value	Description
S.82.20_GM000144	SivK	ACX68641.1	99.341	0	SivK [Streptococcus salivarius]
S.82.20_GM000145	SivR	ACX68642.1	99.569	2.74 × 10^−169^	SivR [Streptococcus salivarius]
S.82.20_GM000146	SivA	ABI54434.1	100	1.51 × 10^−36^	SivA (plasmid) [Streptococcus salivarius]
S.82.20_GM000147	SivM	WP_013990309.1	99.569	0	Salivaricin 9 biosynthesis lanthionine synthetase SivM [Streptococcus salivarius]
S.82.20_GM000148	SivT	ACX68645.1	99.422	0	Putative ABC transporter [Streptococcus salivarius]
S.82.20_GM000149	SivF	EGX29340.1	100	0	Transport ATP-binding protein (plasmid) [Streptococcus salivarius]
S.82.20_GM000150	SivE	ACX68647.1	99.602	2.72 × 10^−179^	Putative transporter [Streptococcus salivarius]
S.82.20_GM000151	SivG	ACX68648.1	100	3.86 × 10^−178^	Putative transporter [Streptococcus salivarius]

## Data Availability

The data presented in this study are openly available from the NCBI in the BioProject PRJNA1215946.

## Supplementary Materials

The following supporting information can be downloaded at: https://www.mdpi.com/article/10.3390/microorganisms13030694/s1, Table S1: Genomes used in the phylogenetic analysis and their respective NCBI accession numbers.
